# Workplace Incivility and Employee Performance: Does Trust in Supervisors Matter? (A Dual Theory Perspective)

**DOI:** 10.3390/bs12120513

**Published:** 2022-12-15

**Authors:** Farida Saleem, Muhammad Imran Malik, Iqra Asif, Awais Qasim

**Affiliations:** 1 Department of Management, College of Business Administration, Prince Sultan University, Riyadh 11586, Saudi Arabia; 2 Department of Management Sciences, COMSATS University Islamabad, Attock Campus, Attock 43600, Pakistan; 3 Department of Management, Lincoln University College, Petaling Jaya 47301, Malaysia

**Keywords:** conservation of resources theory, incivility, Maslow’s hierarchy of needs, performance, trust in supervisors

## Abstract

Employee performance is the backbone of achieving competitiveness and sustainability. This study aims to examine the impact of workplace incivility on employee performance. In addition, trust in supervisors is examined as a mediator. The conservation of resources (COR) theory and Maslow’s hierarchy of needs theory provided the grounds for developing the framework. The data were collected through closed-ended questionnaires and were analyzed using structural equation modeling with SmartPLS. The results affirmed that incivility is harmful to the performance of employees, and that trust in supervisors helps employees to perform well. The trust in the supervisor significantly mediates the incivility–performance relationship. The examination of the proposed model through the lens of two theories as well as the study of low-intensity deviant workplace behavior in a collectivist and developing economy are the contributions of this study to the growing body of literature. However, the use of a single sector was one of the limitations of this study.

## 1. Introduction

Workplace incivility, a low-intensity deviant workplace behavior with an ambiguous intent to harm (Andersson and Pearson, 1999) [1], is noticed everywhere and has become a problem that matters [2]. Harmony and calmness do not always characterize human behavior. Consequently, the occurrence of incivility inside organizations as well as against external stakeholders (customers) is very common [2]. Employees consider behaviors much more meaningful than processes [3]. Employees expect respectful work behavior to achieve organizational outcomes [4]. Moreover, respect is necessary to avoid deviant work behaviors, sustain operations [5], and retain valuable employees [6]. 

Negative factors such as workplace incivility compel employees to hide knowledge from others [7], resulting in harm to an organization’s performance. Incivility adopted by managers harms subordinates’ morale and makes them lose concentration on work [8]. The incivility may decrease the employees’ job satisfaction and push them to leave their jobs [9]. In these situations, trust is the only factor that can keep the employees on track. Trust in the supervisor for his proficiency, experience, and goal orientation may help employees to ignore incivility and do their job.

Organizational behavior literature has started focusing on the negative factors prevailing in the workplace during the past two decades. Several studies have examined the consequences of deviant workplace behaviors at the organizational, group, and individual levels. Schilpzand, Pater, and Erez ([10], p. S57), in their review article about incivility, observed that “the literature mainly focused on topics such as workplace aggression, deviance, bullying, and abusive supervision and predominately investigated the detrimental effects of negative workplace behaviors on targets’ work attitudes, work behaviors, and well-being”, and that workplace incivility—a less intense deviant behavior—is a relatively new addition to the negative behaviors seen at work. Organizational behavior experts have recommended an in-depth examination of workplace incivility [8]. Additionally, Jawahar and Schreurs [8] considered various organizations from different sectors, producing inconclusive results and demanding sector-specific examinations. Similarly, the literature on employee performance tends to focus more on examining factors that count positively toward employee performance [11,12,13], and little evidence exists of stressors that harm employee performance, including incivility.

When it comes to telecommunication organizations, the employees have to deal with many customers with different demands. Therefore, they need a comfortable and peaceful work environment to fulfill these demands. In contrast, a hostile environment accompanied by incivility makes them emotionally and physically exhausted [14], which results in work failures and decreases in performance. Pakistan became the world’s third-fastest-growing telecommunications market in 2008, with the highest mobile penetration rate in the South Asian region in 2018 [15]. The fast-growing telecommunication sector of Pakistan requires an efficient and effective pool of employees; hence, the behavior and performance of employees in this sector are critical.

The aim of the current investigation is two-fold. First, it has not only combined two streams of incivility (coworker and supervisor incivility) affecting employee performance while using trust in the supervisor as an explanatory mechanism, but it has also used telecommunication organizations from a developing economy as a context. Second, the study is conducted in a collectivistic society where people do not expect to receive incivility and believe in maintaining harmonious relationships [16]. Hence, this investigation’s results will help generalize the findings of studies conducted in western, individualistic, and developed countries. 

This paper is divided into five major sections. Section 1 is an introduction to the topic and includes gap identification. This is followed by Section 2, which provides the theoretical framework and review of the relevant literature. Section 3 explains the methods adopted for the analysis. Section 4 discusses the data analysis and the use of SEM, as well as the results. Finally, Section 5 discusses the results, implications, limitations, and possible directions for future research, as well as some concluding remarks. 

## 2. Theory and Hypotheses

### 2.1. Conservation of Resources Theory

The framework for this study is grounded in Hobfoll’s [17] conservation of resources (COR) theory. It is believed that every individual in the organization develops, protects, and retains resources for successful outcomes. These resources may include social relationships, respect, and recognition [18]. Respect is treated as a resource in the collectivist community because trusted employees can be relied upon [19] and help achieve faster decision making [20]. In contrast, losing such resources can induce distress and aggression. These resources in the organization can be increased or decreased, and this fluctuation directly impacts employees’ well-being and performance [21]. Work-related resource depletion is linked to lower empowerment, decreased job satisfaction, and higher intent to leave the organization [22]. 

The theory can also be illustrated in the following way: when everyone in the organization tries to grab the physical/material resources, the push and pull of the resources create unwanted competition. Thus, incivility and reduction in trust occur. This reduces respect for others and harms interpersonal relationships, possibly harming performance. However, respect is a basic human need per Maslow’s hierarchy of needs theory.

### 2.2. Maslow’s Hierarchy of Needs Theory

Maslow’s hierarchy of needs theory has five stages, and each stage is important. The self-respect stage requires respect from everyone present in the organization. It is essential to respect employees as human beings because this will motivate them to perform well, e.g., to concentrate on their work and be engaged, to fulfill responsibilities, to take care of the organizational resources, and to achieve the organizational targets.

**Figure 1:**
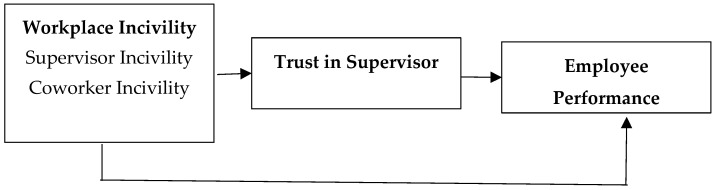
The research framework.

Conversely, the destructive behaviors of supervisors and coworkers harm the respect of the individuals, thus causing them to develop stress due to disrespect. Employees experiencing disrespect tend to avoid hard work and feel depressed, leading to an inability to concentrate on their job due to the negative energy created. They feel uncomfortable in the workplace and try to remain absent from work. Such inconsistencies push them toward decreased performance. The research framework is presented in Figure 1:

### 2.3. Workplace Incivility and Employee Performance

Andersson and Pearson [1] viewed incivility as a low-intensity deviant behavior with ambiguous intent to harm the target [23]. There can be several sources of incivility, such as supervisors and coworkers. Most employees are not shielded from incivility, whether verbal or non-verbal [24]. Supervisors are found to be involved in such behaviors and add to workplace problems instead of reducing them [8,10]. 

Incivility stems from loss of temper, which may be lost due to high work demands. People become insensitive to their coworkers’ work and non-work needs and behave rudely [8]. In contrast, employee performance requires smooth functioning in the workplace [11]. Performance and performance appraisals are interrelated. In the absence of trust in the supervisor, appraisals carried out by others, such as the supervisor, may harm the expectations of the employees and result in decreased performance [25] and may produce incivility among groups. 

By incivility we mean degrading or willingly ignoring others while performing in the workplace and making crucial work-related decisions [8]. A supervisor who behaves badly lowers individuals’ morale and harms their self-efficacy [26], thus harming their work outcomes. Those treated badly try to avoid their supervisor and their colleagues. This avoiding behavior stops them from gathering and sharing the information necessary to carry out their work [10], thus damaging individual and organizational performance [27].

Bad supervisors are a source of stress for employees, creating health problems that further affect the quality of their work life [28]. Managers are found “doing the wrong stuff” due to a lack of the “right stuff” [29]. These managers lose their temper, bully others, behave arrogantly, and ignore the strengths of others [30]. Employees who experience uncivil behavior are likely to develop low energy levels and experience emotional distress. As a result, they lose energy and develop intentions to leave their job [31]. Thoughts about leaving the job also contribute toward decreased performance levels. 

The victims of incivility forego focusing on their performance targets. Instead, they indulge in work-avoidance behaviors, avoiding work as well as the people at work. When they remain away from their work and try to hide by taking unnecessary breaks or leave, they may not be able to do well at work, thus preventing tasks from being completed and harming their discretionary performance [8]. Smidt, De Beer, Brink, and Leiter [32] argued that incivility negatively affects the different groups in an organization. For example, Pearson and Porath [33] found that those individuals who experience incivility in hierarchical relationships become annoyed with their job, abstain from developing innovative products and organizational systems/structures, and misuse organizational resources [24,33]. Moreover, individuals experiencing an unfriendly environment in the workplace remain gloomy, experience nervousness, and are more likely to commit suicide. Similarly, Lim and Cortina [34] (2005) found that vulgar behaviors and incivility, and working in organizations in which these are common, cause harmful effects on performance (Skarlicki & Folger, 1997).

Organizations looking for productive and capable workers must provide realistic conditions that support employee performance. As Mathis and Jackson [35] (2009) revealed, practicality, quality, amount of output, employee participation, and productivity are affected by the behaviors adopted in the workplace, especially negative behaviors. Based on the available evidence, it is hypothesized that:

**H1:**  *There is a negative relationship between a supervisor’s incivility and employee performance*

**H2:**  *There is a negative relationship between coworker incivility and employee performance*

### 2.4. Workplace Incivility and Trust in Supervisors

Negative behaviors negatively affect the employees because they are considered anti-social and can harm relationships [36]. Workplace incivility can be seen at any level of the hierarchy, produces unfavorable working conditions, and damages employees’ learning and development, thus leaving people with limited performance outcomes. An employee’s exposure to incivility may reflect frustration, anger, stressful or aggressive actions [37], and damaged organizational trust [38].

Those who feel that incivility adopted by supervisors is not intended to humiliate others, but to correct actions, may turn evil into good and motivate employees to stay connected with their supervisor and achieve targets [33].

Incivility is associated with stress, and stressed people avoid meeting and greeting others. They frequently become exhausted emotionally and physically [39], and experience decreased job satisfaction [40]. The hypotheses developed are as follows:

**H3:**  *There is a negative relationship between a supervisor’s incivility and trust in the supervisor*

**H4:**  *There is a negative relationship between a coworker’s incivility and trust in the supervisor*

### 2.5. Trust in Supervisors and Employee Performance

Trust in a supervisor is the degree to which employees feel that their supervisor is capable and competent enough to manage organizational resources and operations [41]. Trust is important in order for people see their supervisors as role models and have confidence in them to resolve problems arising during work. A trusted supervisor also has the ability to control organizational resources, including employees. 

It is noted that a “happy worker is a productive worker”. When employees are satisfied with their supervisors and their work environment, they tend to produce more and achieve organizational goals [42]. The trust between various groups working together results in uncountable benefits, e.g., timely decisions, the adoption of organizational change, the saving of organizational resources, and happier employees [43]. The performance of employees is also supported through the sharing of work-related ideas and the seeking of advice from bosses and colleagues working together. Thus, the following hypothesis is developed:

**H5:**  *There is a positive relationship between trust in a supervisor and employee performance*

## 3. Methodology

### Population and Sampling

Telecommunication employees were selected as they have to deal with heavy workloads and customer demands for which well-managed employees and a peaceful work environment are necessary. However, the demands on the employees can make them behave rudely (adopting incivility). A sample of 252 employees was selected. The data were collected using closed-ended questionnaires. 

The scale for workplace incivility (supervisors and coworkers) was adopted from the study of Cortina, Magley, Williams, and Langhout [24]. Employee performance was measured using a questionnaire adapted from Griffin, Neal, and Parker [44]. Trust in supervisors was examined using an 11-item questionnaire from Mcallister [45]. The questionnaire used for data collection is available in the Appendix A. All the items were assessed using a five-point Likert scale. The regions of Rawalpindi and Islamabad (Pakistan) were selected because of the numerous telecommunication company outlets located in these cities, creating a good competitive environment. Social desirability bias was avoided, the questionnaires were kept anonymous, and the confidentiality of respondents was ensured.

## 4. Results

### 4.1. Demographic Information

Basic demographic information about the telecommunication employees is given in Table 1.

The demographic information shows that the employees that participated in the survey had adequate experience and education to understand the statements written in the questionnaires and respond to them accordingly. The employees were asked about the workplace incivility prevailing in their (telecommunication) organizations. Moreover, only male employees took part in the survey. 

### 4.2. Measurement Model

The partial least square (PLS) approach has the strength to test compound relationships simultaneously [46]. Running SEM through SmartPLS requires no fulfillment of assumptions regarding data being normally distributed and accommodated even using small samples [47]. PLS–SEM models provide a good substitute for covariance structural equation modeling [48]. The measurement model is a composition of factor loadings, composite reliabilities, and AVE. All these measures show the degree to which the items used for measurement are in line with the construct. The reliability of the scales and validity of the measures are reported in Table 2.

The scales’ reliability is adequate and above the required value of 0.7, and the values of AVE also meet the adequacy threshold of 0.5. The measurement model looked at the external loadings to determine whether the loading of each statement was adequately loaded on the latent variable. Factor loadings of observed variable are provided in Table 3.

After the removal of the undesired factor loadings (EP3 and WI7), adequate results were obtained. The minimum level of factor loading should be 0.7 or above, as observed in the table. Further, we examined the values of discriminant validity that expressed the extent to which each variable is distinct from the other variables in the measurement model according to theoretical standards. Table 4 shows the square roots of the AVE scores compared with the correlations of the other latent variables. As per standards, the square root of the AVE of each construct should be greater than the corresponding correlation value of the other constructs [49]. 

The values reported in the diagonals are the square roots of the AVE scores. As per Fornell and Larcker’s [49] criteria for the discriminant validity of the variables, the values reported in the column must be lower than the first value reported at the top, and this is fulfilled by the given table.

### 4.3. Structural Model

**Figure 2:**
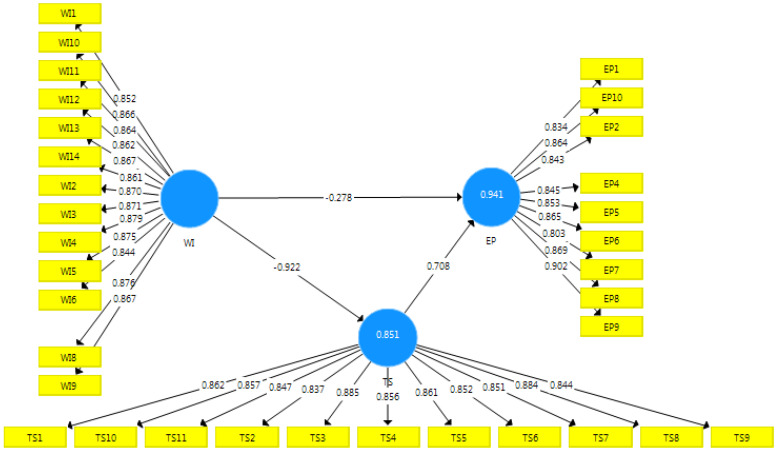
The research framework tested using SmartPLS.

A structural equation model was evaluated using SmartPLS to examine the significance of the path coefficients that show the strength of the relationship between independent and dependent variables. Moreover, the R-square values examined the measure of variance explained by the independent variable. Table 5 shows the path coefficient results and t-values that were determined using a bootstrapping resampling procedure in SmartPLS [50]. A bootstrap procedure with 5000 subsamples was used to determine the statistical significance of the path coefficients. The path analysis is presented in Figure 2 and Table 5.

The results show a positive and significant link between trust in the supervisor and performance (beta = 0.708, *p* = 0.000). Further, it is noted that workplace incivility has a negative and significant relationship with employee performance (beta = −0.278, *p* = 0.000) and trust (beta = −0.922, *p* = 0.000). Finally, the overall workplace incivility is considered collectively, and its relationship is examined.

While looking at the mediation analysis presented in the last row of the table, the results with greater values are found to be significant. In the direct analysis of the relationship between workplace incivility and employee performance, the t-statistics value was 3.918, whereas, after the bootstrapping and the addition of trust as a mediator, it can be easily seen that the t-statistics value has grown to 11.120 with a *p*-value equal to 0.000. This shows that trust in the supervisor has a mediating effect on the relationship. However, unfortunately, due to its negative nature, workplace incivility has converted trust into a negative phenomenon. Further, for detailed analysis, the relationship between supervisor and coworker incivility was added to the model to examine the relationship between trust and employee performance. 

Table 6 shows that supervisor incivility (beta = −0.368, *p* = 0.000) and coworker incivility (beta = −0.569, *p* = 0.000) have a negative and significant relationship with trust. In addition, supervisor incivility (beta = −0.202, *p* = 0.000) has a negative and significant relationship with employee performance. In contrast, coworker incivility (beta = −0.077, *p* = 0.238) has a negative but non-significant relationship with EP. Only trust in the supervisor (beta = 0.712, *p* = 0.000) positively affects employee performance. Figure 2 presents a detailed examination.

### 4.4. Robustness (Nonlinear Effects)

Nonlinearities in the relationships were examined using two tests previously used by Svensson et al. [51]. Firstly, Ramsey’s [52] RESET was performed on the latent variable and the scores were extracted after the convergence of the original model’s PLS–SEM algorithm. Neither the partial regression of the effect of trust in the supervisor on supervisor incivility and coworker incivility (F (3,483) = 0.56, *p* = 0.626) nor the partial regression of the effect of employee performance on trust in the supervisor, supervisor incivility, and coworker incivility (F (3,482) = 1.46, *p* = 0.213) were subject to nonlinearities. Secondly, interaction terms were included to represent the quadratic effects of (1) supervisor incivility and coworker incivility on trust in the supervisor and (2) supervisor incivility, coworker incivility, and trust in the supervisor on employee performance. The results of the bootstrapping with 5000 samples and no sign changes indicate that neither of the nonlinear effects is significant (Table 7). It is therefore concluded that the linear effects model is robust.

## 5. Discussion 

Examining how workplace incivility reduces employee productivity is a significant area of research. Therefore, there were five hypotheses developed for this study. The results of the study support a few of the earlier findings, such as the findings of Jawahar and Schreurs [8], who reported a negative relationship between supervisor incivility in the workplace and employee performance. However, at the same time, the results do not support the premise that incivility or rude behaviors are necessarily required for better employee outcomes [53]. Moreover, it is noted that uncivil behaviors are consistently seen throughout the organizations and are considered a daily routine [54], but that resolving and minimizing these behaviors is as necessary as any other factor for the functioning of these organizations.

The difference between civility and incivility must be clarified here. Civility is the cumulative sum of the numerous sacrifices of living respectfully and has been used from the earliest starting point of civilization as a sign of regard for our fellow residents [24]. Incivility presents an inverse picture of the respect and honor that characterize civility. The opposite of civility is incivility, and it is characterized by humiliation and not giving value to those working at the same workplace. Therefore, there is a need to identify the reason for incivility in the workplace. Moreover, the notion that the weak performance of the employees triggers the supervisors to adopt uncivil behavior is an inadequate premise. It is likely that when workers’ work is hampered due to a few non-performing employees in the workplace, it compels them to behave with incivility toward their coworkers.

The presence of trust can provide several benefits, such as an engaged and satisfied workforce having high levels of organizational commitment, thus enhancing performance outcomes. However, unfortunately, it is noted that the majority (up to or more than 90%) of employees are exposed to uncivil behaviors in the workplace [27,55].

Workplace incivility is harmful if not avoided [10,27]. However, other studies have examined the direct relationships between workplace incivility and employee performance, and only a few have focused on examining indirect relationships such as trust among workers. Therefore, the variables considered for examining indirect relationships are necessary because they help further explain the existing relationships.

The current examination reported that supervisor incivility brings negativity, and that coworker incivility harms at a greater rate. Generally, coworkers engaging in counterproductive and antisocial behaviors leads them to adopt uncivil behaviors to achieve personal benefits. Incivility is responsible for withdrawing employees’ focus from work and drawing their attention toward alternative job options. As a result, they may think of avoiding their job and ultimately adopt the option of leaving their jobs. These results are in line with Beattie and Griffin’s [56] findings, which state that the individuals who are more exposed to workplace incivility have a greater chance of having lower work engagement than those who are less exposed to uncivil behaviors. The results are also supported by the findings of Sharifirad’s [57] work, which has revealed the negative impact of a leader’s incivility on team members’ creativity in organizations. Additionally, the negative relationship between workplace incivility and performance becomes weaker when trust is added to the model.

While looking at the mediating relationship of trust, it becomes clear that trust is an integral part of the workplace because workplace incivility produces burnout related to the job [58]. Workplace trust can make the situation normal and build confidence among groups, settle the existing confusion, and help to lower employee turnover intentions. It is observed through the examination of relationships that there still exist employees in these organizations who trust their supervisor and do not dislike them due to the incivility they adopt. They may like their supervisor due to other factors, such as his capability, benevolence, and integrity. The incivility prevailing in the organization may be due to the integrity factor of the supervisor. Others may take credit for it and use it as a negative tool for adopting incivility [59].

The literature regarding workplace maltreatment has highlighted various factors that resemble workplace incivility, such as various types of harassment [60], social demolition [61], assault [62], and disgust [63]. Various kinds of incivility diminish employee work performance [24]. Incivility from colleagues weakens the targeted individual’s ability to accomplish work [54] (Lim et al., 2008) and increases work-related stress [34], employee turnover intentions [54], and work uncertainty [64]. Incivility from colleagues diminishes the intention among colleagues to be helpful and creates a distance between coworkers that ultimately results in conflicts and diminishes the execution of work [65,66]. 

Moreover, when customers observe incivility occurring in the workplace, they may also adopt uncivil behavior that can be connected with a diminishing of workers’ emotional wellbeing, a component of burnout in which feelings of exhaustion increase, thus stopping an employee from meeting work demands [67]. From the perspective of Maslow’s hierarchy of needs theory, respect is necessary for an employee to keep them motivated and committed. It is possible to avoid performance lags through the development of trust. 

### 5.1. Implications and Suggestions

In telecommunication organizations, the employees deal with different groups, such as coworkers, bosses, and customers. They are required to fulfill new orders, disseminate information to customers, register complaints, and so forth. This requires the provision of a conducive environment for the employees. Therefore, training must focus on inculcating not only the soft skills but also the hard skills of the supervisors and employees. The soft skills training may cover aspects such as communication and personality development skills. In contrast, the hard skills pertain to the systems and software used to deal with the customers and employees. Moreover, time management skills are also beneficial for avoiding mismanagement and incivility in the workplace. Telecommunication companies must focus on tactics to develop employee–employer trust to gain various benefits, such as timely decision making and better administrative control.

To avoid incivility, it is necessary for organizations to train supervisors and employees. This will help companies realize that dealing with employees and customers with incivility annoys them and makes them reluctant to use their services. Telecommunication organizations usually have rapidly dynamic and progressive technological environments with tight schedules and budgets for meeting customer demands. Therefore, appreciating employees and showing them respect can make them more effective. 

Incivility can be avoided by adopting systematic assembly line procedures that help avoid conflicts and interruptions. This will help deal with each customer separately and enhance trust and satisfaction. At the time of recruitment, a balance must be kept when considering employees’ personality traits. Placing blame on others is linked with creating incivility.

The two theories used for the study are the conservation of resource theory and Maslow’s hierarchy of needs theory. The results support the theories developed. According to the resource conservation theory, psychological stress may be caused in three instances: when there is a threat of a loss of resources, when there is an actual net loss of resources, and when there is a lack of gained resources following the spending of resources. Thus, the loss of these resources will drive individuals to certain stress levels. Therefore, individuals try to retain the resources for use to avoid stress. 

According to the conservation of resources theory, the supervisor tries his best to retain the resources for exercising his power and tries to retain his position through his authority over the distribution of resources to subordinates, thus giving him the ability to influence subordinates and show incivility due to the power he holds. The same is true for coworkers who have resources and who work in a sequence, e.g., where the assembly line concept is used. Therefore, the workers may become a source of hindrance to their coworkers’ work and can contribute to the development of an environment of incivility.

In the second theory considered for this study, Maslow’s hierarchy of needs theory, it was assumed that each worker in the workplace demands respect while working in the workplace. If respect is decreased or removed from the workplace, the motivation of workers is reduced, and their work performance will be disturbed. That is why respect is an essential element in organizations that will enable them to avoid any circumstances that damage the performance of employees. Respect is seen as the need of a human being for better work outcomes. If this need is not fulfilled, the individual may not be able to move ahead and accomplish individual or organizational goals.

### 5.2. Limitations and Future Directions

In this study, only a single sector, telecommunications, was considered, thus limiting the results and our ability to generalize to other sectors. Secondly, the questionnaires were distributed to the male and female respondents, but only very few females (seven) took part in the study and returned incomplete survey forms. At the time of data entry and analysis, due to the incomplete data, those responses were removed. This might have an impact on the generalizability of the results. Further, it is suggested that the researchers in their studies consider selecting a bigger sample size for a more complete explanation of the relationships examined. 

Enriching the existing framework by adding variables on either side of the model is recommended. Moreover, indirect relationships can also be examined by adding mediators or moderators to the framework. Possible moderators that can be included in the framework include employee self-efficacy, discipline, and time management.

## 6. Conclusions

This study concludes that employees who try to remain calm and show satisfaction with their jobs tend to perform better than those who are influenced by the destructive behaviors of managers. Incivility in the workplace prevents employees from moving forward in a positive direction, and disrupted trust also hampers the strong bond of relationships. Managers should avoid engaging in abusive behaviors and undermining their employees while working together. Thus, the creation of negative energy in the workplace is optional because it results in the spoilage of resources and the wastage of time and effort instead of contributing positively toward the achievement of organizational goals.

## Figures and Tables

**Table 1:** Demographic information of respondents.

Variable	Category	No. of Respondents	Percentage
Age	26–35 years	43	17.1
	36–45 years	138	54.8
	46–55 years	71.0	28.2
	Total	252	100
Experience	1–5 years	97	38.5
	6–10 years	149	59.1
	Over 11 years	06	02.4
	Total	252	100
Gender	Male	252	100
	Female	00	00.0
	Total	252	100
Education	Bachelor’s	39	15.1
	Master’s	44	17.5
	MS	170	67.5
	Total	252	100

Source: Data recorded from employees.

**Table 2:** Reliability and AVE of the scales.

Variable	No. of Items	Composite Reliability	AVE
Workplace incivility	14	0.944	0.749
Employee performance	10	0.968	0.728
Trust in supervisor	11	0.968	0.736

Source: SmartPLS results.

**Table 3:** Revised CFA (outer loadings).

Item	EP	TS	WI
EP1	0.834		
EP2	0.843		
EP4	0.845		
EP5	0.853		
EP6	0.865		
EP7	0.803		
EP8	0.869		
EP9	0.902		
EP10	0.864		
TS1		0.862	
TS2		0.837	
TS3		0.885	
TS4		0.856	
TS5		0.861	
TS6		0.852	
TS7		0.851	
TS8		0.884	
TS9		0.844	
TS10		0.857	
TS11		0.847	
WI1			0.852
WI2			0.870
WI3			0.871
WI4			0.879
WI5			0.875
WI6			0.844
WI8			0.876
WI9			0.867
WI10			0.866
WI11			0.864
WI12			0.862
WI13			0.867
WI14			0.861

Source: SmartPLS outer loadings.

**Table 4:** Discriminant validity.

	WI	E-Performance	TS
**WI**	0.865		
**E-Performance**	0.595	0.853	
**TS**	0.612	0.639	0.857

**Table 5:** Relationship of the variables.

	Original Sample (O)	Sample Mean (M)	Standard Deviation (STDEV)	T-Statistics	*p*-Values
TS -> EP	0.708	0.702	0.07	10.107	0.000
WI -> EP	−0.278	−0.283	0.071	3.918	0.000
WI -> TS	−0.922	−0.921	0.026	35.167	0.000
WI -> TS -> EP	−0.653	−0.645	0.059	11.120	0.000

Source: SmartPLS results.

**Table 6:** Relationship of the variables.

	Original Sample (O)	Sample Mean (M)	Standard Deviation (STDEV)	T-Statistics (|O/STDEV|)	*p* Values
CI -> EP	−0.077	−0.082	0.065	1.181	0.238
CI -> TS	−0.569	−0.568	0.068	8.42	0.000
SI -> EP	−0.202	−0.207	0.052	3.879	0.000
SI -> TS	−0.368	−0.368	0.066	5.579	0.000
TS -> EP	0.712	0.701	0.070	10.138	0.000

Source: SmartPLS algorithm.

**Table 7:** Nonlinear effects assessment.

Nonlinear Relationship	Coefficient	*p*-Value	f^2^
SI*SI ---> TS	−0.021	0.467	0.001
CI*CI ---> TS	−0.050	0.238	0.005
SI*SI ---> EP	−0.027	0.391	0.002
TS*TS ---> EP	0.012	0.779	0.000
CI*CI ---> EP	−0.060	0.130	0.006

## Data Availability

The data will be available on request from the corresponding author.

## Appendix A. Questionnaire

Workplace incivility and employee performance: the mediating role of trust in supervisors.

**S#**		**Strongly Disagree**	**Disagree**	**Neutral**	**Agree**	**Strongly Agree**
1	**Workplace Incivility** (Cortina, L.M., Magley, V.J., Williams, J.H., & Langhout, R.D. (2001). Incivility in the workplace: Incidence and Impact. *Journal of Occupational Health Psychology*, 6(1), 64–80.) [24]					
**A**	**Uncivil behavior from supervisors**					
WI1	Your supervisor puts you down during at work.	-1-	-2-	-3-	-4-	-5-
WI2	Your supervisor pays little attention to your statements or shows little interest in your opinions.	-1-	-2-	-3-	-4-	-5-
WI3	Your supervisor makes demeaning or derogatory remarks about you.	-1-	-2-	-3-	-4-	-5-
WI4	Your supervisor addresses you in unprofessional terms, either publicly or privately.	-1-	-2-	-3-	-4-	-5-
WI5	Your supervisor ignores or excludes you during meetings, etc.	-1-	-2-	-3-	-4-	-5-
WI6	Your supervisor doubts your judgment on matters for which you have responsibility.	-1-	-2-	-3-	-4-	-5-
WI7	Your supervisor makes unwanted attempts to draw you into discussions of personal matters.	-1-	-2-	-3-	-4-	-5-
**B**	**Uncivil behavior from coworkers**					
WI8	Your coworkers put you down or are condescending to you.	-1-	-2-	-3-	-4-	-5-
WI9	Your coworkers pay little attention to your statements or show little interest in your opinion.	-1-	-2-	-3-	-4-	-5-
WI10	Your coworkers make demeaning or derogatory remarks about you.	-1-	-2-	-3-	-4-	-5-
WI11	Your coworkers address you in unprofessional terms, either publicly or privately.	-1-	-2-	-3-	-4-	-5-
WI12	Your coworkers ignore or exclude you.	-1-	-2-	-3-	-4-	-5-
WI13	Your coworkers doubt your judgment on matters for which you have responsibility.	-1-	-2-	-3-	-4-	-5-
WI14	Your coworkers make unwanted attempts to draw you into discussions of personal matters.	-1-	-2-	-3-	-4-	-5-
2	**Employee Performance** Griffin, M. A., Neal, A., Parker, S. K., (2007), A new model of work role performance: Positive behavior in uncertain and interdependent contexts. *Academy of Management Journal*, 50(2), 327–347. [44]					
**A**	**Individual task proficiency**					
EP1	Carried out the core parts of your job well.	-1-	-2-	-3-	-4-	-5-
EP2	Completed your core tasks well using the standard procedures.	-1-	-2-	-3-	-4-	-5-
EP3	Ensured your tasks were completed properly.	-1-	-2-	-3-	-4-	-5-
**B**	**Individual task adaptivity**					
EP4	Adapted well to changes in core tasks.	-1-	-2-	-3-	-4-	-5-
EP5	Coped with changes to the way you have to do your core tasks.	-1-	-2-	-3-	-4-	-5-
EP6	Learned new skills to help you adapt to changes in your core tasks.	-1-	-2-	-3-	-4-	-5-
EP7	Individual task proactivity.					
EP8	Initiated better ways of doing your core tasks.	-1-	-2-	-3-	-4-	-5-
EP9	Came up with ideas to improve the way in which your core tasks are done.	-1-	-2-	-3-	-4-	-5-
EP10	Made changes to the way your core tasks are done.	-1-	-2-	-3-	-4-	-5-
	Trust in supervisors questionnaire Reference: Mcallister, D.J. (1995). Affect and Cognitive Based Trust As foundations for Interpersonal Cooperation In Organizations. *Academy of management Journal*, 38(1), 24–59. [45]					
**TS1:**	My manager/supervisor performs his/her job with professionalism and dedication.	-1-	-2-	-3-	-4-	-5-
**TS2:**	Given my manager’s/supervisor’s track record, I see no reason to doubt his/her competence and preparation for the job.	-1-	-2-	-3-	-4-	-5-
**TS3:**	I can rely on my manager/supervisor not to make my job more difficult by his careless work.	-1-	-2-	-3-	-4-	-5-
**TS4:**	Most people, even those who are not close friends of my manager/supervisor, trust and respect him/her as a coworker.	-1-	-2-	-3-	-4-	-5-
**TS5:**	My coworkers, who interact with my manager/supervisor, consider him/her to be trustworthy.	-1-	-2-	-3-	-4-	-5-
**TS6:**	If people knew more about my manager’s/supervisor’s background, they would be more concerned and monitor his/her performance more closely.	-1-	-2-	-3-	-4-	-5-
**TS7:**	My manager/supervisor and I have a sharing relationship. We can both freely share our ideas, feelings, and hopes.	-1-	-2-	-3-	-4-	-5-
**TS8:**	I can talk freely to my manager/supervisor about difficulties I am having at work and know that (s)he will want to listen.	-1-	-2-	-3-	-4-	-5-
**TS9:**	My manager/supervisor and I would both feel a sense of loss if one of us were transferred and we could no longer work together.	-1-	-2-	-3-	-4-	-5-
**TS10:**	If I shared my problems with my manager/supervisor, I know (s)he would respond constructively and caringly.	-1-	-2-	-3-	-4-	-5-
**TS11:**	I would have to say that my manager/supervisor and I have both made considerable emotional investments in our working relationship.	-1-	-2-	-3-	-4-	-5-
